# Bibliographical Notices

**Published:** 1855-02

**Authors:** 


### BIBLIOGRAPHICAL NOTICES.

*The Transactions of the American Medical Association. Instituted* 1848. Vol. VII. New York: Chas. B. Norton, 1854.

In our last number, we mentioned the appearance of the volume now before us. We shall at the present time bring before our readers the report "of the Medicinal and Toxicological properties of the Cryptogamic Plants of the United States," by Dr. F. Peyre Porcher, of Charleston, S. C., a gentleman well and favorably known by his previous paper in the 2d Vol. of the Transactions, and as one of the Editors of the Charleston Medical Journal.

It is not our intention to make a critical review of the report, as this could not be done, in a manner at all satisfactory, within the space allotted us. We shall merely take it as a text for a few general observations on the subject, and for pointing out one or two facts, showing the advantages which have accrued to medical science from the study of Cryptogamic botany.

Since the time that hypothetical imaginings as to the causes of the phenomena of nature were recognized as subservient to observation and philosophical experiment, there has been a constant and gradual accumulation of an immense number of facts in every department of physics and Natural History; and science has been continually adding some new and valuable contribution to that rich collection of gifts, which she has already bestowed. The progress of science is in fact, a series of individual discoveries, and the smallest addition to the common treasury of knowledge is a service of inestimable value.

This paper of Dr. Porcher, on the medicinal and toxicological properties of the cryptogamia is especially welcome, as tending to throw additional interest over a department of botany for which we have always felt an especial attachment. The Linnæan maxim "*Natura maxime miranda in minimis,*" nature is chiefly to be admired in the least things, is very appropriately placed at its head. This truth is as yet only partially appreciated, although it is one that should never be lost sight of in the study of nature. If it be philosophical to proceed from the known to the unknown on the pathway of a sound induction, it is equally so to trace the operations of nature in the simpler forms of life, before we attempt the more complex.

The development of the simple before that of the more complicated, appears ever to be the plan of nature, whether her operations be traced in time, or through the dark geological periods of the past. Not only may every animal and plant be traced back to a few simple cells, but the whole organized world is only a series of forms which exhibit the successive stages of their development.

It is well known to naturalists that there are amongst the simplest protophytes and protozoa, plants and animals which are composed of a few cells, or which are even unicellular in their organization. It is here that the animal appears to be blended with the vegetable. It has been said that the animal differs from the vegetable cell in that it possesses the power of motion. But this distinction fails. The spores of several of our fresh water algæ, as for instance *Conferva rivularis*, when first discharged from the plant, move about in the water by means of ciliary appendages during a part of their life, and were actually figured as infusorial animals, in this stage of their development by Ehrenberg, but after a while these little infusoria lose their ciliæ and become transformed into these well known filamentous aquatic algæ. Even chemical analysis fails to aid the naturalist, for cellulose, which was long regarded as peculiar to plants, has been found in the tunics of Ascidians, and in the brain of man. All researches into the limits between animals and plants in this condition of their organization have hitherto completely failed. One fact, however, has been clearly ascertained, viz, that both animals and plants originate from the same starting point, *the cell*, and are developed according to similar organic laws.

There is every reason to believe that the history of the development of vegetation on any barren rock, or newly formed coral island, illustrates those stages by which the earth itself became covered with verdure. The first vegetable denizens of the rocky surface are usually cryptogamous plants, such as crustaceous lichens; these are succeeded by the foliaceous species, and by such mosses as *Polytrichum commune*, *Hedwegia ciliata*, and the different varieties of *Leskia* and *Hypnum*, &c., plants which are of very humble growth, and of exceedingly simple structure, consisting, comparatively speaking, of only a few cells. The oxalic acid contained in the thalli of the lichens, together with the oxygen of the atmosphere, slowly disintegrate the rocky surface, and successive generations of these lowly protophytes finally create a humus which gives birth to a more highly organized vegetation. The higher cryptogamia now make their appearance, Polypodium vulgare, Asplenium trichomenes, Asplenium ebeneum, together with the Saxifrages, Arenarias, Aquilegia Canadensis and other phanerogamous plants. Such appears to be the order of nature—the cellular cryptogamia preparing the way for ferns and flowering plants,—the simple preceding the complex.

That cryptogamous plants are the most ancient inhabitants of the earth; that they existed anterior to the Phanerogamia, and formed, for a long succession of ages, a leading feature in the flora of the antediluvian world, is evident, if we consult the pages of geological history. It is true that the cellular cryptogamia, such as lichens and mosses, are very seldom found in a fossil state; but this is not to be wondered at, when we remember that the preservation of plants in this condition necessarily depends on their structure. The fossil cryptogamia, which have a woody and vascular structure, have, however, been preserved in the greatest abundance.*

Fossil plants are found in the aqueous and stratified formations, which have been divided into three great groups, the Palœzoic, the Secondary, and the Tertiary. The Palœzoic rocks include the Silurian, Cambrian and old red sandstone and carboniferous formations. In the Silurian, Cambrian and old red sandstone we meet with the remains of marine plants, and also a few terrestrial species. In the old red sandstone of Scotland, Miller has detected fucoid ferns, and in the same formation at Oporto, Bunbury has found Pecopteris cyathea, P. muricata, and Neuropteris

* The absence of organic remains in rocks is not always sufficient to enable us to state that these rocks were formed before animals or vegetables existed, since the late Prof. Forbes has shown that, even in the present day, there are depths in the ocean which are destitute of organic life. Hence rocks deposited at such depths might contain no organic remains.

tenui-folia, ferns which are closely allied to those of the carboniferous period. There was land, therefore, as well as water at this remote epoch, although the abundance of fishes and marine plants seems to indicate that the sea covered the greater part of the earth's surface.

Towards the close of the palœzoic period, however, land plants appear to have been developed on an enlarged scale. Coal owes its origin to the abundant vegetation of this era; for it is now universally admitted that this substance is of vegetable origin. This the microscope has fully demonstrated. In some kinds of coal, punctuated woody fibre has been detected, in others dotted and scalariform tissue, as well as cells of various kinds. The occurrence of dotted and scalariform vessels indicates the presence of ferns and their allied forms, such as sigillaria, stegmaria and lepidodendrons, whilst true punctuated wood implies the presence of coniferæ.

In the secondary formations we meet with numerous coniferæ, and cycadeæ, whilst ferns and lycopodiaceæ are less abundant and not so gigantic in their growth. The tertiary period is characterised by an abundance of angiospermous dicotyledons, and of monocotyledons, more especially of palms. In the flora of the most recent tertiary deposits, we meet with coniferæ, rosaceæ, legumenosæ, &c., and with a small number of dicotyledons with gamospetalous corollas. There appears to have been a similar progression in the animal creation. Thus fishes and reptiles preceded the development of mammalia, and for innumerable ages appear to have been the predominating form.

In what we have now said we have attempted to show that the operations of organic law are the same both in the animal and vegetable world; that in both kingdoms there is a *regular gradation* from the simple, or imperfect, to the complex, or more perfect.

Few, we believe, recognize as they ought to do the benefits which have already occurred to physiology, and the science of medicine from the study of a few humble cryptogamia. The great cell-doctrine of physiology, which is now admitted to be the basis of all sound scientific investigations into the phenomena of organized beings, originated in the study of vegetable matter. M. Mirbel, in a most admirable memoir on the development of

*Marchantia polymorpha*, a little acotyledonous plant belonging to the family of the *Hepaticæ*, was the first to show the cellular origin of every other form of vegetable tissue. He proved that the fibre cells of plants are only attenuated utricles, and that the different varieties of vasiform tissue and ducts, by which the interior of the plant is aerated, originate in a row of utricles ; these gradually elongate on their internal surface, and the various secondary deposits characteristic of the different forms of spiral vessels, gradually make their appearance ; the septa or partition walls between the several cellules are then absorbed, and the transformation of the utricles into vessels is completed. These observations were confirmed by the researches of Schleiden and other distinguished botanists, and thus a flood of light was thrown on the organization of plants. This discovery may thus be succinctly stated. The primitive form of all cells is that of a closed spherical vesicle or utricle. There is no plant or organ of a plant which is not at the commencement of its growth fabricated *exclusively* of cells, which approach more or less to that of a sphere in form. It is, however, only in plants which are very low in organization such as algæ and lichens that the cells remain in this condition ; in vegetation of a higher grade this uniformity speedily disappears, and the individuality of the cells becomes manifest as growth progresses. While some of them continue spherical, others take a much higher degree of development and become gradually transformed into woody fibre and spiral vessels.

But how do the cells of plants originate ? Whence come those new utricles which without ceasing are added to those already in existence, and which augment incessantly the mass from whence they draw their origin ? These are difficult, but exceedingly interesting questions. The philosophical botanist has sought for their solution amongst cryptogamous plants, and his labors have been abundantly rewarded. A German naturalist, Mohl, selected for observation one of the fresh water algæ, which had been previously figured and described as *Conferva glomerata.* This simple thread-like plant was placed beneath the microscope, and the development of the row of utricles of which its entire organization consists, watched. Very soon, Mohl observed that the interior face of the cavity of one of the utricles presented towards its middle part a fold, which increased almost imperceptibly until it ended by forming a complete wall dividing the cavity of the utricle into two parts. Each of these then dilated itself into a new utricle. Thus in the place of one cell there were two cells, which again divided in the same way, and so on. It is in this way that a single cell gives rise to a row of connected cells, when the division takes place in one direction, and to a plane or solid mass when it takes place in two or more directions. There are other modes of increase which we have not space to enumerate,—suffice it to say that their discovery originated in the investigation of cryptogamous plants of extreme simplicity of organization.

Up to this period it was believed by the most eminent physiologists that animal and vegetable tissues differed widely in their developement; and that cells existed only in plants. Such was the condition of things in 1838, when Schwann, taking up the beautiful investigations that Schleiden had just published upon the structure and growth of vegetable cells, came to the conclusion that animal tissue consisted equally of cells, and that whatever may be the character of the tissues, whether they assume the form of muscle, bones or bloodvessels, all originate in cells of which they are but modifications. The cells which form the tissue of the higher animals in their earliest condition, present the same uniformity of appearance in their external configuration as the cells of plants; some of them retain this cellular condition throughout the life of the animal. Thus what is called adipose tissue consists of a mass of globular or dodecahedral cells containing fat in their interior. Others, however, rapidly undergo a change of form in accordance with those laws of growth to which they are individually subject, and assume the form of bone, cartilage, bloodvessels, &c. In this respect precisely the same laws govern both the animal and vegetable world.

We find some further illustrations of an instructive kind, in the minutiæ of organic structure and embryology. It has been already stated that the basis of all vegetable and animal substances consist of cells. Nutriment is converted into these before being assimilated by the system. The tissues are formed from them. The ovum destined to be a new creature, is originally a cell with a contained granule. We see it acting this reproductive part in the simplest manner in the cryptogamic plants.

"The parent cell, arrived at maturity by its exercise of its organic functions, bursts, and liberates its contained granules. These at once thrown upon their own resources, and entirely dependent for their nutrition on the surrounding elements, develop themselves into new cells, which repeat the life of their original. Amongst the higher tribes of the cryptogamia, the reproductive cell does not burst, but the first cells of the new structure are developed within it, and these gradually extend, by a similar process of multiplication, into that primary leaf-like expansion which is the first-formed structure in all plants."* Here we see the little cell becomes directly a plant, the full formed living being.

These grand discoveries which have given such an impulse to medical science originated in botanical investigations into the nature of cryptogamic vegetation, in minds which appreciated the truth of the Linnaæn maxim, "*Natura maxime miranda in minimis.*"

The examination of cryptogamic plants, with reference to their medicinal and toxicological properties, has been prosecuted abroad with considerable success; and Dr. Porcher, in his Report, has added to his own labors what has been accomplished elsewhere.

In conclusion, we cannot express the very high opinion which we have formed of this paper better than by adopting the language of an able writer in another place: "This is an able and valuable paper, one which has evidently cost its author a vast amount of labor. To bring together, as he has done in this report, the leading facts that have been developed by various observers in relation to the properties and probable available uses of the cryptogamous plants met with in this country; to indicate their respective localities, and to identify their species and varieties, so as to be able correctly to appropriate the recorded observations in relation to their curative, poisonous or other properties, was, we can readily conceive, a task of no trifling character, demanding considerable research, much time and no little patience, more especially as the entire subject belongs to a department of enquiry which has had few laborers in this country."

We shall take another opportunity of examining the other Reports published in the present number. At present it may suffice

* Carpenter's Report on the Results obtained by the microscope in the study of Anatomy and Physiology, 1843.

to say that we consider them as very creditable productions, quite equal, if not superior to their predecessors in other volumes. One word before we conclude, as to the manner in which the present volume has been published. We should have made no remark on this had not the subject been forced upon our notice by the unmeasured praises that have been bestowed upon it by one or more of the New York journals, and the unfair comparisons that have been made between the present Committee of Publication and their predecessors, in respect to the manner in which they have have performed their respective tasks.

We confess that we can discover no reason for the laudation that has been so profusely bestowed upon the committee having charge of the publication of the volume before us. The paper upon which it is printed is, we admit, of an excellent quality, while its typographical execution is as good as need be desired. In these respects the volume will compare very favorably with either of the preceding. If this can be viewed as a subject of commendation, we very cheerfully congratulate the committee upon it

We must, however, deny to the latter the credit that has been claimed for them from the circumstance of all the subscribers to the present volume of Transactions being furnished with copies bound in cloth and lettered, without any additional charge. This, it is to be recollected, was done in accordance with a recommendation of the former Committee of Publication, submitted to and adopted to by the Association at its last session. It is much to be regretted that this recommendation had not been fully complied with, and the volume before us made to correspond, in size and lettering, with the preceding volumes that were supplied to members in the same style of binding. This would have obviated the mortification which many now experience in being obliged to have upon their shelves a series of volumes of the same work, one of which is cut down nearly half an inch in the length of its page below the others, while the lettering upon its back is entirely different in its style and arrangement.

We have heard it said that the defect just alluded to may readily be overlooked in consideration of the much earlier period at which this volume of the Transactions has been issued than several of the preceding ones. We are well convinced that there has been no unnecessary delay in the publication of the present volume; its appearance has not, however, been any earlier than that of the preceding ones. It embraces just two hundred and one pages less than the volume for 1853; it contains no wood cuts, and but four easily executed lithographs, in addition to those which accompany the paper of Dr. Brainard; which latter were engraved in Paris in anticipation, we suspect, of the presentation of the essay they are designed to illustrate. The volume of the preceding year, besides its greater bulk, contained twenty-four lithographic drawings, the majority requiring considerable time for their proper execution, and sixty-seven wood cuts. It was, nevertheless, published only one week later than the present committee, with all their efforts, were able to issue the volume of Transactions committed to their charge.

The contents of the volume are clumsily arranged. The index to the valuable paper of Dr. Porcher is inserted altogether out of place, being at the very end of the volume, after the general index of the latter. Various other inaccuracies might be pointed out. Thus, the prize essay of Dr. Brainard is accredited on both its titles to Dr. David Brainard, whereas it is the production of Dr. Daniel Brainard. The list of permanent members is replete with errors, some of them altogether inexcusable; while the very important resolution of Dr. Atlee, adopted at the last session of the Association, requiring the Constitution with its several amendments to be printed with each volume of the Transactions, has been either overlooked or entirely disregarded by the committee.

We are perfectly willing to make allowance for many of the defects in the execution of the volume before us. The task of edit ing and publishing a work similar to these Transactions is certainly a difficult and laborious one, and when committed to those who have neither sufficient experience or leisure for its proper execution, cannot fail to be more or less imperfectly performed. We should have been among the last to notice the short-comings of the present Committee of Publication, had not the unwise and fulsome commendations of their friends, and the disparaging remarks bestowed by them upon the preceding committee, in order to magnify the labors of the former, seemed to render an examination of their pretensions necessary and proper.

*A Manual of Pathological Anatomy.* By C. HANDFIELD JONES, M.B., F.R.S., &c.; and EDWARD H. SIEVEKING, M.D., &c. *First American Edition, revised. With three hundred and ninety seven illustrations.* Philadelphia: Blanchard & Lea. 1854.

We cordially welcome the above manual as a most valuable addition to the literature of the subject of which it treats. "The absence of any original work in the English language which embraces the whole subject" of pathological anatomy—the apology offered by the authors for its composition—greatly enhances our obligations for its acquisition. The necessity of an intimate acquaintance with the various morbid processes which affect the human organism need not, here, be spoken of. A just appreciation of these and of the effects produced by them should be considered indispensable to the practitioner both of surgery and medicine. The possession and application of such knowledge constitute, in fact, one of the essential differences between the accomplished physician, and him whose guidance is, at best, but mere conjecture.

The first part of the work, nearly one third of the whole book, is devoted to the subject of General Pathological Anatomy. Under this head, after some excellent preliminary observations, are described: Functional Derangement; Morbid States of the Blood; Textural Changes; New Formations—Tumors; and the Parasites existing in the Human Organization. The Pathological Anatomy of the Nervous System; of the Organs of Circulation; of the Organs of Respiration; of the Alimentary Canal; of the Urinary Apparatus; of the Female Organs of Generation; of the Joints; and, finally, of the Osseous System are then successively treated of.

Dr. Jones' observations regarding the immunity afforded by certain affections against tuberculous disease are both striking and instructive.

"Cystic growths are not often associated with tubercle, and the same is true of cancerous; when the latter are present together with tubercle, they are, in most cases, of secondary origin to it. Rokitansky contrasts the frequency of tuberculization of the lungs with the rarity of pulmonary cancer; the frequency of ovarian, gastric, and rectal cancer, with the rarity of tuberculous deposit in these parts. These and other facts indicate that the one morbid process tends to exclude the other. Typhus and the exanthemata, he states, do not commonly attack the tuberculous, but they are very apt to be followed by tuberculous disease. Sufferers from intermittent fever, goîtrous disease, and rachitis, seem to be, *pro tanto*, less liable to tuberculous affection. The non-coexistence of aneurismal and tuberculous disease depends, in Rokitansky's opinion, on the exhaustion of the fibrinous constituent of the blood, by the deposits taking place on the inner surface of the sac. An especial immunity against tubercle is afforded by an abnormally venous condition of the blood, from whatever cause this may come to pass. Congenital malformations of the heart or great blood vessels; morbid alterations of the same; deformities of the chest, producing contraction of its cavity; annihilation of the function of one lung by pleuritic effusion; abdominal growths, preventing the free descent of the diaphragm; chronic pulmonary catarrh; emphysema and bronchial dilatation, have all been observed as exercising an unquestionable counter influence against the development of tubercle; and in all these conditions the free oxygenation of the blood is more or less interfered with. The undoubted effect of pregnancy in delaying the advance of tuberculous disease of the lungs, is explained by Rokitansky on the same principle of impeded, and consequently imperfect respiration, inducing a venous condition of blood; and he refers to the great production of fibrin, which takes place after parturition, as confirmatory of this view—tubercle being regarded as a fibriniform product."

As relating to the same subject, we would, here, draw attention to the following statement, (page 424:)

"A species of antithesis exists in the lungs between the tendency to tubercular deposit in their apices and the liability of the lower lobes to idiopathic inflammation. This may be accounted for by the encouragement to deposition from the blood offered in the apices of the lungs, by the lesser expansibility of these parts. The movement of the clavicle and two upper ribs is very small compared with that of the lower ribs, and the intercostal muscles are more rigid here than below, circumstances that are enhanced by a sluggish habit, while they favor any accidental impediment to the aeration of the blood. This, in its turn, diminishes the rapidity of the current as well as its quality; hence the morbid deposition ensues, and, as the cavities of the air-vesicles are the points of least resistance, the effusion is effected into them. We have already mentioned that the more rapid the course of phthisis, the more generally we find the deposit diffused through the lung. In a case of acute miliary tubercle, from which one of our illustrations is taken, the bases and the apices of both lungs were uniformly affected; both organs being studded throughout with the deposit, which had probably taken place but a few days previous to death, in a subject debilitated by rheumatic affection of the heart and central softening of the brain."

The conflicting views which are held regarding the value of the microscope as a positive means of distinguishing cancerous from other tumors, as well as the widely different opinions respecting their curability by operation or otherwise, are strongly evidenced in the discussion now going on before the Parisian Academy of Medicine. It will be seen by the following extract that Dr. Jones agrees with M. Velpeau in his very moderate estimate of the microscope as a means of diagnosis of such structures. After mentioning the different forms of cancer generally met with, Dr. Jones remarks:

"There may be, probably, other varieties of cancerous tumors, or, to put it otherwise, tumors possessing more or less of cancerousness; but we have now sketched the outline of the principal forms that are usually met with, and we feel convinced that it is far more important for the student and the practitioner, to contemplate steadily the great characteristics of cancerous disease, than to load his memory with the details of the incidental and trivial. Partly on this account we have not attempted to give any very minute description of the structure of cancerous tumors, for our own examinations have most thoroughly convinced us of the non-existence of any special structural character, absolutely and in all cases distinctive of cancer. This point, which is in accordance with the teachings of the best authorities, seems far from being correctly understood in the present day, and we cannot but think that there is still much tendency to over-estimate the microscope as a means for the diagnosis of cancer. It is our opinion that the cases are very rare indeed, where the microscope will avail to detect cancer with any certainty, where the naked eye features are insufficient. On the other hand, we have more than once seen unquestionable cancers made up of substance which we should have been led, from microscopic examination alone, to consider as of a simple nature."

The opinion of the same writer upon the curability of cancer by operation, though guarded, is, on the whole, unfavorable to such a proceeding, excepting as regards the epithelial varieties of the disease. He says:

"In concluding this subject, we may offer a few remarks with reference to the effects of removing cancerous tumors by operation. In the first place, it is quite clear that the disease is manifestly constitutional, and that no sound, real cure can be expected from merely removing its external development. Secondly, it is matter of experience, that in not a few instances surgical interference with one tumor has provoked the speedy appearance of several others. Thirdly, any attempt at removal is useless; nay, may be absolutely injurious, unless every particle of cancerous structure is taken away. Fourthly, epithelial cancers seem least prone to return after removal; encephaloid invariably does, and mostly with great rapidity; scirrhus may be checked in its progress, but its return can very rarely be prevented. The check which may be given by operation to the progress of cancer depends on the circumstance before stated, that a tumor, once formed, becomes an instrument for the multiplication of similar tumors and intensification of the diathesis. It must require a combination of favorable circumstances, or a great intensity of the diathesis, to insure the development of effused blastema into an heterologous growth; but when this has taken place, then the very growth and vital actions of the structure will constantly generate fresh supplies of cancerous blastema, and thus promote the formation of secondary cancers. The destruction, therefore, of the growth, which thus reacts so evily upon the system, may be reasonably expected, *if it do not aggravate,* to delay the cause of the disease. But the misfortune is, that, as above stated, it does sometimes aggravate, and that fearfully, a previously indolent cancerous diathesis. Dr. Walshe says, 'excision of a tumor seems to awaken a dormant force, cancers spring up in all directions, and enlarge with a power of vegetation almost incredible.' Why this should happen, we do not know; but we may conjecture that when the original diathesis is slight, the formation of a tumor may tend in some degree to localize it, and leave the system in a somewhat healthier state, provided the tumor itself be chiefly fibrous, and produce but a small amount of blastema. The removal of the indolent tumor may be analogous to the cure of fistula in ano in a person of phthisical tendency. The two principles referred to of the cancerous tumor, in one case acting as a cause, increasing the force of the disease, and in another retarding it, are not contradictory, though opposite; they will prevail in different degrees in different instances, according to the kind of tumor and other circumstances."

Our limited space alone restrains us from noticing more at length the various subjects treated of in this interesting work. Presenting, as it does, an excellent summary of the existing state of knowledge in relation to pathological Anatomy, we cannot too strongly urge upon the student the necessity of a thorough acquaintance with its contents.

---

*Address delivered before the Philadelphia County Medical Society.* By Thos. F. Betton, M.D., M.A.N.S., President of the Society, Fellow of the College of Physicians of Philadelphia. Dec. 13th, 1854. Published by the Society.

It is perhaps not altogether becoming in the members of a society, albeit behind the reviewer's shield, to trumpet the praises of their own President's official production. We do not propose, therefore, to alarm his modesty, or any body's sense of propriety, by needless though natural gratulations on the very happy and elevated, as well as practically useful manner and matter of Dr. Betton's address. Still, simple truth requires us to say that we have fairly read it through, as we had previously listened to it, with unusual interest and pleasure, and a considerable degree of pride. Those who have enjoyed the same privilege must agree to the justness of this estimate, and we entertain no fear of a different result with others who may have the benefit of its perusal yet in store for them.

All will admit that the sentiments are those of a genuine lover of his calling, and of one who has the best and only true interests of his brethren at heart; and we are not ashamed to acknowledge full allegiance to the spirit of such an emanation from the highest seat of our professional synagogue. Any enumeration, however, of the reasons for the favorable impression here expressed, would only lead to an undesirable transgression of the rule of conduct under which we have just set out. Several questions are touched upon in such a way as strongly to incline us to occupy, in extracts, an amount of space which is already otherwise absorbed; others again are suggested which, in these days of agitation and progress, it is hard to avoid discussing. Such passages, for instance, as the following, and more of similar import:

"How, then, shall we begin to restore the palmy days of our profession, and see them shine with the same refulgence that illuminated them in the days of Rush, Shippen, Wistar, Physick, and a host of other departed worthies? How shall we exterminate quackery and all its attendant evils? Not by opposition, nor by ridicule, nor by satire, nor even by legislative enactments, were it possible to obtain them—for, like the rebel weed, it grows most luxuriantly when most trampled upon—but by elevating the tone and standard of ourselves, and of those who are to succeed us. Let us actively set about and demand a radical improvement in the course of medical instruction in this country; not by extending the term of lectures, nor by increasing the number of subjects now taught, but by exacting a more thorough preliminary education of those who enter the portals of our medical schools. Any one who mixes much among his medical brethren must be pained to find the lamentable ignorance displayed, by many, of all the branches of polite learning, so necessary to form the character of a physician and a gentleman. Let it be required of all who present themselves as pupils, to possess, at least, a correct knowledge of their own language, and such attainments in the classics as to enable them to construe the easier authors; or, at all events, to write correctly the common apothecary Latin of the day."

Our chief object in the present notice is to invite particular attention to one of the most important topics treated by the orator. It is a topic, too, which possesses a virtue decidedly rare in these annual demonstrations—novelty. We refer to the management of what are mis-called the College Clinics. Dr. Betton's remarks upon the evils of the system now in vogue among the schools, are so entirely in accordance with the views maintained in the January number of this Journal, and sustain them so directly, that we cannot deny ourselves the satisfaction of quoting them at length.

"The plan of clinical teaching in our medical schools, specious as it may at first sight appear, and attractive, as it certainly is, to the newly initiated student, anxious to witness, for the first time, the shedding of human blood, or wonder at the coolness and dexterity of the surgeon, is, at best, but little better than an advertisement. It does not teach operative surgery, for that can be learned practically only in the dissecting-room on the subject. It diverts clinical teaching from its proper channel of hospitals, almshouses and infirmaries, and is a direct injury to the profession at large, more especially to the junior members of it, by monopolizing practice which would otherwise fall under their hands. Who would not, very naturally and wisely, prefer submitting to a surgical operation at the hands of distinguished professors, eminent for their experience and skill, rather than to be subjected to the knife of a tyro, no matter what may be his talents and qualifications: particularly when all these advantages are accompanied by the magical inducement, that the relief is afforded *gratuitously*. It is underselling others, since many resort to the public clinics, who would willingly and readily afford some compensation, thereby taking this from the pocket of some deserving and aspiring young man, to whom, in all probability, it would have been of importance; and depriving him, moreover, of the opportunity of gaining that skill and experience which would give him high rank in his profession. In bygone days students never received any public clinical instruction in the amphitheatre of the schools; yet, were it allowable, we could point out as many physicians as learned, and surgeons as dexterous, as any who have graduated under the modern and so-called improved system. That it attracts pupils, and increases the size of the class, I believe there can be no doubt; this, perhaps, is all that is required. That it fails to instruct the pupil in an adequate degree, and that it is unjust to the profession generally, I believe there can be equally none."

We felt satisfied that the Examiner, in commenting on the evils and abuses of the so-called clinical demonstrations at our medical schools, was only giving active expression to a deeply felt conviction of the mass of our associates in practice, and of not a few even of the teachers of medicine. We knew that its language was but a faint echo of the long burthened and too long unuttered thoughts of the great majority of those around us, whose opinions, in such questions, in order to be conclusive in their operation, need only to be followed by decisive and united action. It was, nevertheless, a consummation far beyond our hopes to meet, at the very first step, with the frank and vigorous support of one whose position, at the head of those who are most aggrieved, because most extensively subjected to the wrong, gives peculiar significance and weight to his official warning.

We sincerely trust that the members of the body to whom that warning was addressed will not let it pass without appropriate response and a corresponding effort in the right direction. They have the power, and sooner or later they will be forced in self-defence to exercise it. They have been sleeping in a blissful ignorance, while a paralysing incubus was daily growing in their midst. They have discovered, just in time, that an overgrown monopoly in the guise of charitable science was threatening, under shelter of its delusive pretensions and advantages, to darken, if not destroy, their prospects of advancement.

Let us not be understood, in complaining of one portion of the prevailing system of practical instruction, to be approving of another portion, which, as at present frequently administered, may be scarcely less faulty and insufficient. Nor could we pretend, in agreeing with the proposition to double the established number of clinical lessons at the hospital, to do more than advocate it as the least possible step towards reform, and simply because that step is not only immediately practicable, but immediately demanded by the crying necessities of all concerned.

Until a better state of things is obtained in this country, we would be glad to see an entire separation between the elementary and didactic exercises of the lecture room and the practical exhibitions of disease, its results and its management at the bed-side. There is quite enough to do in both branches of teaching to afford abundance of legitimate work, during the customary term of study, to two distinct corps of preceptors. Whether the European example be followed in this respect or not, it is very certain that the attempt to convert a class-room into a sick-ward, an operating theatre, or even into a consulting dispensary, during two hours of two days in the week, cannot fail to produce a mongrel institution, which, as a whole, meets few of the real wants of the occasion, while it does endless injury by substituting a mockery and sham for a substantial good to the student on the one hand and the patient on the other.

We may return to the hospital curriculum at some future period. Our business now is with a far more potent evil. We have to deal, not with the *bonâ fide* clinical instruction imperatively needed by the student to prepare him for his destined work in life, but with its miserable counterfeit presented in a system which, producing a hotch-potch of the worst features of the hospital and the dispensary together, succeeds in imitating neither well enough to reconcile us with the absence of the other.

The important question is, how far does the objectionable system fail in meeting the acknowledged requirements of the learner?

We have no wish to under-rate the legitimate uses of a dispensary. The prescribing room of such a charity or the out-patients ward of a hospital, certainly afford an excellent field for a valuable amount and kind of practical training. But the student must diligently delve in it with his own hands; he must follow a competent adviser step by step in the greatest possible number and variety of cases; and, although at length familiarized with, but still studying, the walking specimens of human suffering, he must look within the hospital and watch long and earnestly among the fractures, the serious wounds, the fevers, the multiform disorders and stages of disease that occupy the beds, before he begins to think his student work accomplished.

The solid advantages obtainable from a Dispensary for the practical investigation of slight, incipient and other forms of portable disease and injury are unquestionably great, although we do not agree that such classes of medical and surgical disorder constitute a materially large proportion of the young practitioner's every day experience. In the amphitheatrical "clinic," however, the custom of presenting showy operations and attractive lectures, during the two hours a week devoted to each branch of practice, effectually does away with the main and sole recommendation to the out-patient plan, as a means of practical improvement.

Out of the whole number presenting themselves for professional treatment, a small fraction only can be exhibited, or dressed or operated on before the class; and these few are to be gazed at from a distance, if not through an ever waving mass of bushy heads and jutting shoulders. What becomes of the remainder, and who see and prescribe and otherwise attend to them, it is not for us to say. They doubtless enjoy the usual share of tender mercies for such cases made and provided, and undoubtedly may claim the honor of facilitating the practical improvement of the few enterprising votaries of science who find their way behind the scenes. But the majority of the lookers on in the class room know and learn nothing from the crowd of cases that are hastened through by the professor or his clinical assistant, during the brief time allowed for consultation and advice.

Unfortunately, again, we meet a stumbling block which might be easily forgotten by every one except the really practical inquirer. Who is to insure a second visit from the patient whose interesting ailment has been once provided for? And who is to guarantee that he will adhere to any one of the directions with which he has been formally dismissed in the hearing of the class? It is unnecessary to pursue this subject further. We need no ghost from the grave to tell us how to estimate the scientific value of the absurd performances which are thus dignified with the title and importance of lessons in the healing art. But the great boast—the leading card of these displays is their surgery—their splendid operations!

Here, at least, is no mistake, and something tangible! It is visible sometimes, and audible, but rarely tangible. Even in the vision must the faith be constantly in service. The vaunted operation, the crepitating bone, the projecting dislocation, the fluctuating abscess, the impermeable stricture, may each be brilliantly portrayed to the willing ear, and trustingly believed in; but he is a happy man indeed whose admission to the sanctum sanctorum of the arena has enabled him to touch or even see distinctly what is so fluently described; so, too, in many operations, the excited student must very often be content with faith, as the only evidence of the things not seen. Such and many similar difficulties, are, in short,inseparable from the plan adopted. They have been briefly hinted at without the remotest intention to reflect upon the truthfulness of the honorable and able teachers who are involved in these unsatisfactory and delusive exhibitions. Some of these objections, of course hold good against the hospital as well as the dispensary, especially so far as the demonstrations of the theatre are concerned. The worst of the dispensary is, that there is no help for such defects so long as the functions of a hospital are assumed, and the true and only province of the out-ward is abandoned or exceeded.

We have nearly exhausted our allotted space, but have barely entered on our subject. Occasions will not be wanting for a return to it again. We may then have something to say, amongst other matter, in relation to the moral effects of the system of gratuitous advice so shamefully abused in these clinics. This view of the question affords ample material for a chapter without allusion to other considerations. It has been for some time past, the sole burthen of numerous leaders and a multitude of letters in the British periodicals, under the impulse of an agitation in the mother country, which we earnestly hope will be attended with permanently good results,—results that may save us in America from the same kind of quicksands, towards which the members of the profession in this and other countries have been good naturedly and most blindly drifting. Let us once clearly open our eyes to the tendency of our own supineness, and nothing will be easier than to avert the threatened danger.

